# Role of immunometabolism during congenital cytomegalovirus infection

**DOI:** 10.1097/IN9.0000000000000034

**Published:** 2023-11-28

**Authors:** Kevin J. Zwezdaryk, Amitinder Kaur

**Affiliations:** 1 Department of Microbiology and Immunology, Tulane University School of Medicine, New Orleans, LA, USA; 2 Tulane Center for Aging, Tulane University School of Medicine, New Orleans, LA, USA; 3 Tulane Brain Institute, Tulane University School of Medicine, New Orleans, LA, USA; 4 Division of Immunology, Tulane National Primate Research Center, Covington, LA, USA

**Keywords:** placenta, immunometabolism, congenital cytomegalovirus

## Abstract

Cytomegalovirus (CMV) is a master manipulator of host metabolic pathways. The impact of CMV metabolic rewiring during congenital CMV on immune function is unknown. CMV infection can directly alter glycolytic and oxidative phosphorylation pathways in infected cells. Recent data suggests CMV may alter metabolism in uninfected neighboring cells. In this mini review, we discuss how CMV infection may impact immune function through metabolic pathways. We discuss how immune cells differ between maternal and decidual compartments and how altered immunometabolism may contribute to congenital infections.

A healthy pregnancy results from temporal adaptations involving physiologic, metabolic, endocrine, microbiotic, and immunological networks. Physiologically, there is increased circulating blood volume, heart rate, cardiac function, and respiratory tidal volume. Metabolically, nutrient requirements are increased to meet the biosynthetic and bioenergetic demands of the fetus ^[1]^. A shift from an energy-storage anabolic state (elevated fat deposition and lower blood glucose) in the first two trimesters of gestation to a catabolic state (lipolysis and blood glucose concentrations increase) occurs in late pregnancy. The role of sex hormones has been well-defined during gestation. The gut microbiota influences physiology and directly modulates the endocrine and metabolic systems. Together, the physiological, metabolic, and endocrine systems establish a homeostatic environment that is supportive of a healthy pregnancy. Critically, the homeostasis of the immune system is tightly interconnected with these factors. The signals from the mother, fetus, and placenta must be coordinated and integrated with each system. As pregnancy is not a static event but changes throughout gestation, each system must adapt interdependently during gestation. An integrated understanding of the local and systemic factors influencing this network will provide clarity regarding healthy and pathological pregnancies.

Central to this discussion are immunometabolic adaptations during pregnancy. The immune system must establish tolerance for the fetus and maintain effective function against pathogens. Dysregulation of immunological adaptations is hypothesized to be a key factor driving adverse pregnancy outcomes, including preeclampsia and intrauterine growth restriction ^[2]^. Increased inflammation after implantation is mediated through the effects of placental hormones and the corpus luteum and is characterized by an increase in myeloid cells, a decrease in lymphocytes, and altered lipid maintenance. How metabolic and immunological changes during pregnancy influence the progression and resolution of maternal viral infections has not been adequately defined. Viruses may perturb immunological adaptations during pregnancy, resulting in increased disease severity, as has been reported for SARS-CoV-2, hepatitis E virus, Zika virus, Ebola virus, and cytomegalovirus (CMV). Insight into how viruses disrupt local and systemic homeostasis during pregnancy may be closely linked to metabolism. Viruses are obligate parasites that depend on host metabolic machinery to meet the bioenergetic and biosynthetic demands of replication ^[3]^. CMV is an optimal example. During replication, CMV has been shown to increase glycolysis, glutaminolysis, and the tricarboxylic acid cycle (TCA). Mitochondrial electron transport chain (ETC) activity and oxidative phosphorylation (OXPHOS) are essential for CMV replication ^[4,5]^. CMV is a common species-specific betaherpesvirus that typically results in a life-long persistent asymptomatic infection in an immunocompetent host. CMV is the most common viral cause of congenital infection, complicating 40,000 births in the United States annually ^[6]^. Up to 25% of infants born with congenital CMV (cCMV) will have permanent disabilities, including hearing loss, cognitive and motor impairments, and seizure disorders ^[7]^. In utero CMV transmission occurs in 33%–50% of pregnant CMV-seronegative women with primary infection and in 1.4% after nonprimary infection in pregnant CMV-seropositive women ^[8]^. Despite the lower transmission rates, regions with high CMV seroprevalence account for a major part of the global burden of cCMV ^[9]^.

Studies in humans and mice have shown that CMV has profound effects on the innate and adaptive immune system ^[10]^. Studies in monozygotic twins discordant for CMV infection have shown CMV to be a major driver of nonheritable influences on immune maturation ^[11]^. Nearly 50% of children in the United States are seropositive for CMV. It is unknown how early in development CMV may imprint on the immune system. How maternal CMV infection during pregnancy affects fetal immune development, regardless of direct fetal or placental infection, remains unclear. The effect of CMV infection on the immune system may be a direct consequence of its effect on cellular metabolism. Metabolism shapes the differentiation, activation, and function of immune cell subsets. HIV infection has been correlated with changes in immunometabolism and has been linked to chronic immune activation and CD4^+^ T cell depletion ^[12,13]^. We are using the novel nonhuman primate (NHP) model of cCMV infection in rhesus macaques ^[14]^ along with studies of normal pregnancy in rhesus CMV-seronegative and CMV-seropositive macaques to understand the effects of primary and chronic CMV infection on immunometabolism at the placental-maternal interface.

Upon encountering a pathogen, immune cells differentiate into effectors needed for a successful defense. Immune cells must alter metabolic phenotypes to meet the large biosynthetic and energetic demands this requires. Many immune cell phenotypes use glucose through glycolysis for growth and survival, regardless of the availability of oxygen ^[15,16]^. This allows glycolytic metabolites to be used for biosynthetic purposes, including lipid, amino acid, and hexosamine (protein glycosylation) precursors ^[17,18]^. Memory T cells rely on OXPHOS for longevity and effective function ^[19]^. During restimulation, memory T cells utilize both glycolysis and OXPHOS to respond to recall antigens ^[20–22]^. A hybrid glycolytic/OXPHOS metabolic phenotype supports the rapid functional response characteristic of memory T cells. Glycolytic flux is increased with CD8^+^ T cell activation. The increased flux can be measured starting as early as 15 minutes after T cell receptor (TCR) stimulation. Prolonged increases in glycolytic flux depend on CD28 co-stimulatory signaling. Glycolysis is used to meet the increased energy demand through lactate production. It also provides intermediates to drive the TCA cycle and produce biosynthetic materials (lipids, nucleotides, and proteins) required for T cell proliferation, differentiation, and effector function. As viruses upregulate similar pathways following infection, CD8^+^ T cells may be in direct competition for glucose and glutamine. Mitochondrial and glucose metabolism dysfunction in CD8^+^ T cells has been observed in a chronic hepatitis B virus model ^[23]^. In HIV-infected individuals, the CD4^+^ T cells have elevated glycolysis, including increased GLUT-1 expression, hexokinase, and lactate levels ^[13]^. HIV-infected CD16^+^ monocytes also display an elevated glycolytic phenotype, including increased GLUT-1 expression, glucose uptake, and lactate levels ^[24]^. This is different from quiescent cells, which display an OXPHOS phenotype. These examples highlight the importance of immunometabolism in immune cell function and fate during acute and chronic viral infections.

The NHP pregnancy model provides a unique opportunity to study placental immunometabolism in normal pregnancy and in the setting of an experimental congenital infection. Rhesus macaque placentation resembles humans in consisting of a hemochorial placenta with trophoblast invasion of the uterine wall ^[25]^. In a comprehensive immunophenotyping analysis of the rhesus macaque maternal-fetal interface in normal pregnancy across gestation, we showed key similarities in the composition of decidual leukocytes between rhesus macaques and humans ^[26]^. Macaque decidual leukocytes are enriched for activated effector memory and tissue-resident CD4^+^ and CD8^+^ T lymphocytes, natural killer (NK) cells, and monocytes/macrophages that are phenotypically different from those in peripheral blood (Figure 1). In an experimental model of congenital Zika virus infection, we detected changes in the decidual leukocyte composition suggestive of local immunosuppression with a reduced frequency of activated, cytotoxic, CXCR3^+^ memory T lymphocytes ^[26]^. A study of viral-induced metabolic changes in the placenta and their impact on antiviral immunity at the maternal-fetal interface will provide valuable insight into local immune determinants of congenital infection.

**Figure 1.**
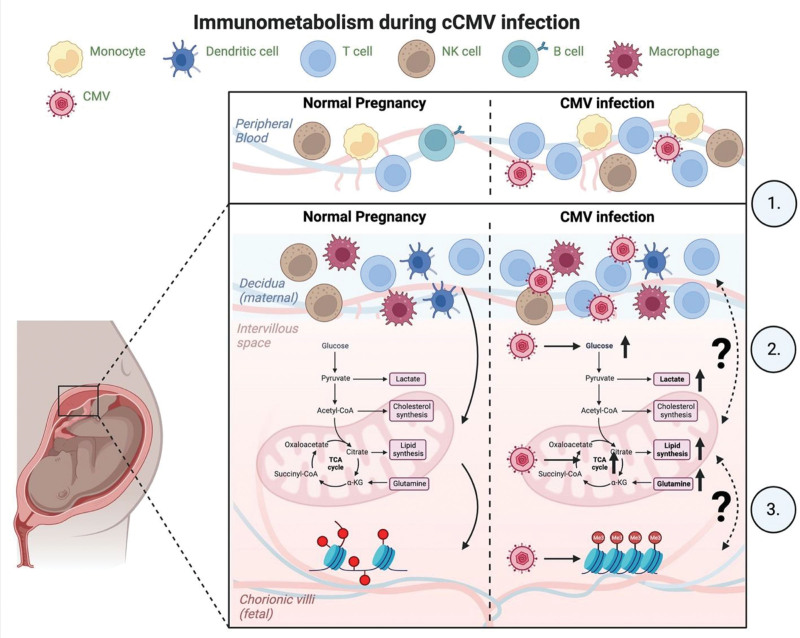
**Schematic overview of immune phenotypes and proposed model of changes in immunometabolism during pregnancy and congenital CMV infection.** In normal pregnancy, the immune repertoire differs when comparing peripheral blood and the decidua. (1) During cCMV infection, there is increased immune activation in the peripheral blood and decidua. In the decidua, there is an enrichment of immune cells, that are phenotypically distinct from peripheral blood. (2) For immune cells to function correctly, immune cell-specific metabolic changes must occur. Many of the required metabolic pathways are manipulated during CMV infection. It is largely unknown if CMV-mediated metabolic changes can alter the metabolic microenvironment, possibly impacting immune function. (3) Metabolites influence the epigenetic landscape. It is unclear if CMV-mediated metabolic changes influence epigenetic status. Together, these factors may dictate viral transmission across the placenta and severity of infection.

There are several mechanisms by which CMV could alter immunometabolism and upset the homeostatic balance in pregnancy (Figure 1). CMV exhibits a wide tropism, including for immune cells. CMV infection may directly compete for metabolites in the microenvironment, decreasing concentrations around infected cells due to increased consumption. Increased glucose uptake by infected cells could starve neighboring cells of glucose, making them more susceptible to CMV infection or limiting the variety or magnitude of response. Many immune cell fates and functions are dependent on increased glycolysis. Reducing available glucose pools could be an anti-immune strategy employed by CMV, resulting in weakened immune responses (Figure 1). Alternately, metabolites or soluble factors released from infected cells may metabolically or epigenetically alter cells in the microenvironment. Glioblastoma cells infected with CMV have been shown to increase glycolysis and temporarily alter epigenetic markers in uninfected, co-cultured fibroblasts ^[27]^. A recent publication described how CMV exploited intercellular signaling to reshape the microenvironment and enhance viral dissemination ^[28]^. It is unknown whether altered metabolism in infected cells can affect immune cells that respond to the viral infection. A sensorineural hearing loss model may provide insight to this question. Pediatric sensorineural hearing loss is the most common permanent complication. Antiviral treatment has been shown to improve hearing status ^[29]^. Treatment efficacy is reported to be influenced by antiviral dosage. Increased dosage has been associated with neutropenia. Decreased dosage decreases antiviral toxicity but may reduce antiviral efficacy. A recent report examined the role of novel pharmacological therapies as synergistic combination therapies. Using a murine model of CMV-associated hearing loss, poloxamer 188 (P188) and quercetin were shown to improve hearing outcomes ^[30]^. A mechanism of action proposed to explain this result was an interaction with the mitochondria and antioxidant properties. Both excipients have been reported to localize to the mitochondria ^[31]^. It is possible that the antiviral activity of the excipients interferes with the mitochondrial function required by CMV for efficient replication, resulting in a decrease in damage to the ear. Alternatively, the drugs may target macrophages and reduce ROS production ^[32]^. Immunopathology due to excessive ROS is a plausible but unvalidated mechanism. This example does not address initial infection but again illustrates a possible role for metabolic targeting during cCMV to improve patient outcomes.

In summary, exploration of how CMV metabolically alters immune cells in the context of pregnancy will shed valuable insight into immune determinants of placental transmission. The functional effects of immunometabolic signaling on immune differentiation and efficacy and the duration of these effects are being considered. The impact of metabolites under these conditions on epigenetic imprinting on fetal immune cells is also being explored. A deeper understanding of these factors in the context of congenital infection will provide insight into targeted therapies and parameters for a healthy pregnancy.

## Conflicts of interest

The authors declare no conflicts of interest.

## Funding

This project is funded by NIH-NICHHD R01HD107790 to A.K and K.Z. and the NIH P51OD011104 to TNPRC.
